# Implementing Facilitated Access to a Text Messaging, Smoking Cessation Intervention Among Swedish Patients Having Elective Surgery: Qualitative Study of Patients’ and Health Care Professionals’ Perspectives

**DOI:** 10.2196/17563

**Published:** 2020-09-18

**Authors:** Kristin Thomas, Marcus Bendtsen, Catharina Linderoth, Preben Bendtsen

**Affiliations:** 1 Department of Health, Medicine and Caring Sciences Linköping University Linköping Sweden

**Keywords:** mHealth, mobile health, text messages, health care, smoking cessation, patients with elective surgery, implementation

## Abstract

**Background:**

There is strong evidence that short-term smoking cessation before surgery can reduce postoperative morbidity. There are, however, several structural problems in health care systems concerning how to implement smoking cessation interventions in routine practice for preoperative patients.

**Objective:**

This study aimed to analyze the implementation of a text messaging, smoking cessation intervention targeting patients having elective surgery. Implementation of facilitated access (ie, referral from practitioners) and the perceived usefulness among patients were investigated. Elective surgery is defined as scheduled, nonacute surgery.

**Methods:**

A qualitative study was carried out at two medium-sized hospitals in the south of Sweden. The implementation of facilitated access was investigated during a 12-month period from April 2018 to April 2019. Facilitated access was conceptualized as specialists recommending the text messaging intervention to patients having elective surgery. Implementation was explored in terms of perceptions about the intervention and behaviors associated with implementation; that is, how patients used the intervention and how specialists behaved in facilitating usage among patients. Two focus groups with smoking cessation specialists and 10 individual interviews with patients were carried out. Qualitative content analysis was used to analyze the data.

**Results:**

Two main categories were identified from the focus group data with smoking cessation specialists: *implementation approach* and *perceptions about the intervention*. The first category, *implementation approach*, referred to how specialists adapted their efforts to situational factors and to the needs and preferences of patients, and how building of trust with patients was prioritized. The second category, *perceptions about the intervention*, showed that specialists thought the content and structure of the text messaging intervention felt familiar and worked well as a complement to current practice. Two categories were identified from the patient interview data: *incorporating new means of support from health care* and *determinants of use*. The first category referred to how patients adopted and incorporated the intervention into their smoking cessation journey. Patients were receptive, shared the text messages with friends and family, humanized the text messages, and used the messages as a complement to other strategies to quit smoking. The second category, *determinants of use*, referred to aspects that influenced how and when patients used the intervention and included the following: timing of the intervention and text messages, motivation to change, and perceptions of the mobile phone medium.

**Conclusions:**

Smoking cessation specialists adopted an active role in implementing the intervention by adapting their approach and fitting the intervention into existing routines. Patients showed strong motivation to change and openness to incorporate the intervention into their behavior change journey; however, the timing of the intervention and messages were important in optimizing the support. A text messaging, smoking cessation intervention can be a valuable and feasible way to reach smoking patients having elective surgery.

## Introduction

Smoking is responsible for more than 60 diseases and is the single-most important preventable factor for disease and premature mortality [1]. In persons between 45 and 64 years of age, the proportion of daily smokers is higher than for any other age group: 10% for women and 9% for men [2]. When including occasional smokers, the proportions are 16% for women and 13% for men. Although the proportion of smokers in Sweden is less than in many other countries, tobacco is associated with 9.6% of the total disease burden [3]. Thus, around 6400 people die every year in Sweden due to smoking.

The negative impact of smoking on outcomes following elective surgery is well established [4-6]. Several large studies have shown that the risk of cardiovascular, respiratory, and wound-healing complications and even death within 30 days of operation is greater for smokers than nonsmokers [7,8]. For instance, one study showed that smokers (11.3%) had a considerably increased risk for postoperative complications compared to nonsmokers (7.5%), especially an increased risk for pulmonary complications. In fact, smoking was found to be an independent risk factor exemplified by the fact that smokers needed intensive care and prolonged postoperative hospital stays to a greater extent than nonsmokers [9]. Research also show that complications can be avoided even with short-term perioperative smoking cessation [10,11]. Findings in a Cochrane review, based on indirect comparisons and evidence from two small trials, show that interventions beginning 4-8 weeks before surgery and including weekly counseling were most likely to have a significant impact on complications and on long-term smoking cessation [10].

However, more knowledge is needed on how to organize smoking cessation support within health care systems, especially for preoperative patients. Research has shown that various factors determine how and to what degree smoking cessation is implemented in routine care. For instance, motivation, knowledge, training, and confidence among health care professionals to deliver smoking cessation support have been shown to have an influence [12-14]. Beliefs and preconceptions among health care professionals that question the value or benefit of smoking cessation support have also been reported; for example, some health care professionals believe that interventions are time-consuming, ineffective, or intrusive [15]. Furthermore, organizational challenges, including lack of standardized pathways or referral routines across and between parts of the health care system, have also been quoted [12]. Indeed, organizational solutions as to how to best implement smoking cessation support for this patient group is unclear, for example, whether smoking cessation support optimally is delivered in hospitals, via specialist care, or in primary care. Although primary care could be a useful setting for smoking cessation support before surgery, challenges to achieve working referral routines have been reported, including long waiting times, resistance from staff in primary health care to offer support to patients from specialist care, and unclear communication between specialist care and primary health care [16,17].

These hurdles to implement smoking cessation support in health care present a need for new types of innovations [17]. One way forward could be to give smokers having elective surgery access to support through their mobile phones. Mobile phone–based interventions that use text messaging typically encompass a series of automated messages during a period of about 8-12 weeks. Messages aim to guide and support participants to plan and prepare for a quit attempt and then reinforce and support continued smoking cessation [18].

There is a substantial body of evidence demonstrating the effect of text messaging on smoking cessation among adolescents [19-21], university students [22], and adults [18,21,23,24]. Furthermore, mobile phone–based interventions including text messaging have shown to be one of the most cost-effective interventions for tobacco control and are endorsed by the World Health Organization [25]. Major advantages have been shown to be cost-effectiveness, reach, and flexibility, whereby they can be delivered at any time [26].

In our previous research, we have developed evidence-based interventions that use text messaging: the NEXit (Nicotine Exit) and NEXit Junior trials [22,27]. These interventions are based on behavior change theory and evidence-based practice guidelines [28,29]. This study aimed to analyze the implementation of a text messaging, smoking cessation intervention targeting patients having elective surgery. Specifically, implementation of facilitated access (ie, referral from practitioners) and the perceived usefulness of the intervention among patients were investigated. Elective surgery is defined as scheduled, nonacute surgery.

## Methods

### Design

In this qualitative study, we investigated the implementation of facilitated access and perceived usefulness of a text messaging, smoking cessation intervention among patients having elective surgery. Two focus groups with smoking cessation specialists and 10 individual interviews with patients were carried out. The implementation of facilitated access (ie, referral from practitioners) was investigated during a 12-month period from April 2018 to April 2019. Implementation was explored in terms of perceptions about the intervention and behaviors associated with implementation; that is, how patients used the intervention and how specialists behaved in facilitating usage among patients.

### Setting

The study was carried out at two medium-sized hospitals in the south of Sweden. The smoking cessation offices were located at the hospitals and received patients by referral from surgeons and walk-ins. Referral came primarily from general, orthopedic, heart, and gastrointestinal surgery departments as well as, to a smaller extent, from pulmonary medicine, rheumatology, and cardiac clinics. Practice routines typically include an initial session with a patient where motivation to quit is explored, followed by follow-up meetings and/or telephone calls depending on the preferences of the patient.

The three smoking cessation specialists were asked to facilitate implementation by inviting patients to sign up for the intervention during a face-to-face visit. Sign-up was done by patients sending a text message with a specific code to a dedicated telephone number. The text messaging, smoking cessation intervention is an evidence-based, 12-week, fully automated text messaging program that has been described elsewhere [30].

In total, 100 patients were approached, and 30 patients signed on to the intervention during the visit, 44 patients wanted to think about signing up at a later stage at home, whereas 26 patients were not interested in the intervention. After signing up to the intervention, the patients received a text message with a link to a baseline questionnaire; a total of 27 patients completed the questionnaire and were thereafter enrolled in the intervention.

### Data Collection and Participants

#### Smoking Cessation Specialists

The smoking cessation specialists were registered nurses with training in smoking cessation counseling. The training included a 3-day course to become registered smoking cessation specialists as well as ongoing supervision. All specialists were also qualified in motivational interviewing. All three smoking cessation specialists were invited and took part in focus group interviews. Data collection was conducted at two time points: at 6 and 12 months after starting to implement the intervention among their patients. The focus group interviews lasted approximately 1 hour; they followed a semistructured format and included questions on the experience of implementing the intervention in routine practice.

CL and PB took part in the first focus group and CL and KT took part in the second focus group. Participants were all women between 45 and 54 years of age.

#### Patient Interviews

Telephone interviews were conducted with patients 3 months after the intervention. A semistructured interview guide was used that aimed to capture patients’ experiences and use of the intervention.

A total of 14 patients were contacted via telephone and invited to take part in interviews; 4 patients declined to participate. A total of 10 interviews were carried out and they lasted between 10 and 40 minutes. Respondents were between 45 and 70 years of age; the sample was made up of 6 men (60%) and 4 women (40%). Telephone interviews were either scheduled later or conducted straight away depending on patients’ preferences.

### Data Analyses

All focus group discussions and individual interviews were audio-recorded and transcribed verbatim. The two datasets—patients and specialists—were analyzed separately. The analysis process, which followed conventional content analysis guidelines by Hsieh and Shannon [31], was used to analyze the data. Conventional content analysis is a structured process where data relevant to the study aim are coded and categorized. First, all transcripts were read through. Second, words and text that depicted areas relating to the study aim were identified (coding). In a parallel process, these identified codes were grouped (categorizing) based on similarity in content and their relation to each other. The content of each category was expanded by revisiting the data and comparing data across formed categories. Lastly, a comparison across categories was done to make sure that categories were defined and described in a way that maximized internal homogeneity and external heterogeneity. KT performed initial data analysis, which was then discussed by KT and PB.

### Ethical Approval

The study has received ethical approval by the Regional Ethical Review Board of Linköping University, Sweden (2018/4-31).

## Results

### Focus Group Interviews With Specialists

#### Overview

Two main categories were identified in the data that described the implementation of the intervention in routine smoking cessation practice: implementation approach and perceptions about the intervention. Initial coding resulted in a number of codes. Examples of these codes are given in Table 1. For example, prioritizing trust referred to how specialists chose to behave, communicate, and give information to patients that enhanced patients’ trust. For instance, specialists actively chose to communicate the intervention at the end of sessions when initial trust between patients and themselves had been established.

**Table 1:** List of categories, subcategories, and code examples for specialist data.

Category and subcategories		Code examples	Excerpts of quotes from raw data^a^
**Implementation approach**			
	Adapting		
		Time	“Sometimes I need to help them, but it is difficult to have enough time.”
		Patient qualities	“[Some patients are] thorough and want to follow the program exactly, exemplary, and want to be best in the class so to speak.”
		Patient capacity	“We have some really old...if we are to talk about age, for old people it is really difficult [to enroll].”
	Trust building		
		Relationship to patient	“Yes, I usually have the same method; I try to create a good relationship with them in the first meeting.”
		Prioritizing trust	“You have to focus on what’s important and get that relationship first that you don’t do by pressuring them.”
**Perceptions of the intervention**			
	Familiarity		
		Compatible with existing routines	“I think it’s been ok to include in the session, like it hasn’t been odd, it’s been easy.”
		Recognizing content	“And it is so much that you recognize in the text messages...so much and similar to what we use in our practice, so that’s good.”
	Complementary		
		Add-on	“No, I’ve seen it as a something additional to what I do normally.”
		Introducing at the end	“I think, also, that I introduce it at the end like the typical session talk first and then at the end introduce it.”

^a^Translation from Swedish to English for the purposes of this publication only.

#### Implementation Approaches

##### Overview

The category *implementation approach* refers to how specialists implemented the intervention and strategies that they used: adapting to specific situations and building trust with patients before introducing the intervention.

##### Adapting

Adapting refers to how specialists altered their behavior and approach to facilitate implementation through, for instance, how they introduced the intervention, talked about it, or assisted the patient in signing up. Adaptations were influenced by patient needs and capacities as well as situational factors. For example, the patients were perceived to have varied capacities and capabilities to sign up and engage in the intervention. One respondent described how one patient was not able to sign up for the intervention due to feeling overwhelmed by the technical requirements of completing the registration and, thus, needed additional support.

When I sat with my patient and together completed the registration with him, he thought “What’s this?” and in the end I had to take the phone and, like, do it for him.

Similarly, the specialists used strategies to invite patients to sign up for the intervention depending on the needs and preferences of the patients. For example, specialists could help patients sign up and complete baseline questionnaires if this would enable patients to start the intervention.

It, you start with the patient, you always have the patient in mind, so there’s lots of different needs for whoever sits there.

Furthermore, adaptations were made due to situational factors such as time constraints. Respondents expressed that it was sometimes challenging to have enough time to introduce and sign up patients within one session. Adaptations were made regarding how they spoke about the intervention or delayed introducing the intervention until later sessions to make sure they would have enough time to engage patients in registration. Finally, one respondent expressed that when meeting patients that were “good student”–type people, it was easier to introduce the intervention and that this group of patients needed less guidance.

Yes, I think about the lonely people, it becomes a friend in a way, that somebody sends you text messages. And those who are really, some are very thoughtful and careful and want to do the right thing always.

##### Trust Building

Respondents expressed that they tried to build trust with patients first and then, often at the end of the session, introduce the intervention. Building trust entailed creating a trusting relationship with the patient by listening to their needs and pacing how much, and what kind of, information they shared with the patient. Respondents expressed that it was useful to invest in this relationship first and then introduce the intervention.

Yes, you can say that I have a way, you try to create a relationship with the patient at the first meeting and then finish with “Oh, we have this study,” and then I tell a bit more about that.

#### Perceptions About the Intervention

##### Overview

The category *perceptions about the intervention* refers to how specialists perceived the intervention: whether its content and structure were familiar, and whether the intervention was a complement to current practice.

##### Familiarity

In general, the respondents were positive toward the intervention and voiced that the concept, content, and structure of the intervention was in line with techniques that they already used in patient practice. Specialists expressed that that they experienced the intervention as familiar, for instance, that its content was straightforward, logical, and compatible with smoking cessation strategies that they already employed among patients. The respondents further described the intervention as feasible and easy to implement. In addition, respondents spoke about mobile phone–based interventions in general and expressed an interest and ease to incorporate these types of tools in practice.

And there’s much, you recognize a lot that’s written in the messages. We got them all on paper before, it is, you recognize, a lot that I use in my work, so I think it is good.

##### Complementary

The intervention was perceived as a complement to be offered to patients alongside existing support and tools, such as follow-up phone calls or medication. The intervention was described as something that was introduced to most patients at the end of the routine smoking cessation session. Respondents perceived the intervention as a complement to what they already did, something parallel that did not necessarily affect established routines but a tool that was feasible to use.

No, but I have seen this as a complement to what I do, so doesn’t disturb what I do, I haven’t seen it in that way.

It’s been an add-on to what we already offer, now we can offer this as well: “Would this be something for you?”

It feels like it’s not what you want to start with; you have to focus on the main issue and create the relationship, and you can’t do that if you pressure the patient.

Furthermore, the intervention was described as a valuable alternative tool when there were limited resources, particularly for patients with complex needs.

Sure, we have some quite intensive patients that need more regular contact and follow-up visits. And then it feels nicer if they have accepted this, to know that, yes, they get something; meanwhile, if I can’t book them in next week when I was supposed to, then I know that they will get some sort of support.

### Individual Interviews With Patients

#### Overview

Two main categories were identified in the data: incorporating new means of support from health care and determinants of use. See Table 2 for examples of these codes.

**Table 2:** List of categories, subcategories, and code examples for patient and specialist data.

Categories and subcategories		Code examples	Excerpts of quotes from raw data^a^
**Incorporating new means of support from health care**			
	Being receptive		
		Curiosity	“I was waiting on the text messages every day, like so like you looked and wondered what have they written today.”
		Openness	“So it was like a bit, ok, I’ve got [a message] from them again [laughs], so it has been really good I think.”
	Sharing		
		Impact beyond user	“I have sent screenshots to a friend when it has been good stuff.”
		Communicating	“And we said, my wife always asked, ‘Is it the smoking cessation people?’ Yes, and now they write this and that.”
	Humanizing of text messages		
		Expectations	“To start again when they have put so much effort in, I felt it would be to let somebody down if I started again.”
		Presence	“It feels like there is somebody sitting and sending the messages.”
	Text messages as a complement		
		Alternative strategies	“So I could do it at my pace and then, and then my wife said it doesn’t matter if it takes 10 minutes or half hour, ‘Let’s go for a walk.’”
		Combination	“I think it was the combination, ‘cause I don’t think text messages alone would do it for me.”
**Determinants of use**			
	Timing		
		Relevance	“Yes I think then that...the text messages are really good when you’re in the middle of it before surgery, then you get triggered.”
		Timing	“But as I had already quit, then it was a bit stupid, this about preparing for quitting in two weeks, then I had already quit.”
	Motivation to change		
		Decisiveness	“Yes, I can see now, I have thought that there is no point or meaning to start again.”
		Action	“And then I walked down to her at the unit straight away from meeting with the surgeon to speak with her.”
	Perceptions of the mobile phone medium		
		Reached	“It was a bit personal, as it came from a text message rather than an app.”
		Limited interaction	“I didn’t go on the links...you got to write why you quit...but I read all the text messages.”

^a^Translation from Swedish to English for the purposes of this publication only.

#### Incorporating New Means of Support From Health Care

##### Overview

This category referred to how patients adopted and incorporated the intervention into their smoking cessation journey. Incorporating new means of support from health care encompassed the following: being receptive, sharing the text messages with friends and family, humanizing of text messages, and using the text messages as a complement to other strategies to quit smoking.

##### Being Receptive

Being receptive refers to having an openness to learn, to assimilate information, and to try techniques suggested in the text messages. Learning is talked about in terms of gaining knowledge about new things, such as how to manage abstinence but also about being reminded of things you already know; for example, the health risk of smoking. Respondents describe an openness to this learning process and that it was useful in their behavior change.

In addition, being receptive was illustrated by curiosity and interest among respondents regarding the content of the messages. This can further be exemplified by a respondent that described that they waited for the text message to arrive in the morning, being eager to know what it would say. The data further showed that users read all the text messages, either when they arrived or later the same day if it was more convenient. Even respondents that expressed irritation from text messages described that they still read all the text messages and reflected on their content.

It is difficult to say. Now I waited for this...every day...like, you looked, “What did they say today?”

##### Sharing

Part of processing the content of the intervention entailed sharing it with significant others. Respondents expressed that they read the text messages and shared what they learned or discrete messages with friends and family. For example, one respondent described that they, together with their partner, each day read the messages and reflected on their content. In this way, using the intervention was a shared activity, similar to the actual smoking cessation journey where family members would be described as an important influencer. Other respondents described that they forwarded messages to friends they knew smoked and who could benefit from the content.

I have a friend that also wants to quit, so I, like, sent the messages to her, the good ones, so I forwarded those to her, like.

##### Humanizing of Text Messages

Respondents spoke about the text messages as personal and compatible with human support. Respondents described that receiving text messages made them feel less lonely, safer, and as if somebody cared about them. For example, one respondent perceived the text messages as a presence, as somebody who cheered them on and encouraged them to keep being smoke-free. Another respondent highlighted nonsmoking norms in society and how smokers are often a minority and that the text messages made him feel less abandoned.

Yes, I think so. Because I think that many people feel like this, that they feel lonely in all of this, like when they do it.

No, I don’t know, but I was really grateful that I got support and that somebody cared and that I wasn’t alone with this to struggle with.

Furthermore, there were similarities between the expectations that respondents placed on the text messages and people in their social environment. For example, one respondent compared the text messages to a nagging relative that wanted them to quit smoking. Similar to expectations in their social environment, this respondent expressed that they did not appreciate nagging but preferred encouraging messages.

Yes, a bit, I have told my family, my friends as well, that “Stop nagging, it doesn’t work.” I think it is even worse then.

##### Text Messages as a Complement

Respondents used the intervention as a complement to other smoking cessation strategies, such as medication and physical activity. As respondents were recruited by the smoking cessation specialist, some had continued support from the specialist, while others did not. Some respondents tried medication or nicotine patches with varied outcomes. Finally, other respondents described finding their own strategies to cope with abstinence, such as going for regular walks. Nevertheless, the text messaging, smoking cessation intervention was described as one piece of the jigsaw that was combined with all the above strategies.

Well...now when I was really motivated ‘cause I had to stop, so I think that I could have done it without the text message, but it was a good complement to the sessions.

#### Determinants of Use

##### Overview

The category *determinants of use* refer to aspects that influenced how and when patients used the intervention and included the timing of the intervention, motivation to change among patients, and perceptions of the mobile phone as a medium for support.

##### Timing

Timing referred to how well the intervention could respond to the needs of the patients. The timing of content contributed to how the intervention was perceived and used among patients. When the timing was optimal, a text message could offer support, consolidate or confirm feelings or states of the users, and empower respondents to keep going. However, when timing was suboptimal, text messages could prompt adverse experiences, such as irritation or craving. For example, one informant described that stopping smoking was not an issue or difficult to do, but that the intervention reminded him about cigarettes and prompted cravings. Another respondent described that she had already stopped smoking when she signed up for the intervention, which made the text messages about preparing to quit have limited relevance for her.

I will be totally honest; I didn’t have any problems quitting at all. But then I got lots of text messages and then I got reminded of it and I started to feel and think, “Do I fancy a cigarette?” So it was the opposite for me. Do you know what I mean?

##### Motivation to Change

The data showed that patients having elective surgery had a strong motivation and persistence for smoking cessation, which contributed to the openness toward using the intervention. There was an urgency to stop smoking among the respondents that stemmed from their health status or requirements from the surgery department. This strong motivation was illustrated by immediate action to stop smoking after having been given the information about surgery or diagnosis. One informant described that they finally had been given a reason to quit the cigarettes, and when compared to previous attempts to stop smoking, this time quitting was effortless.

And then I walked down to her at the unit straight away from meeting with the surgeon to speak with her. And then I said, “Let’s throw away the cigarettes.”

However, the data also showed that motivation to stop smoking could be vulnerable and predominantly depend on surgery scheduling and outcome. For example, one informant described that they had to have multiple surgeries and that they timed their smoking cessation accordingly.

The respondents also took an active role in their care plan. Minimizing risks for complications after surgery were described as a team effort where the respondents acknowledged their responsibility to be smoke-free before surgery. Respondents described their appreciation about having access to surgery and felt that they needed to contribute to optimal conditions for surgery. For example, one respondent explained that she felt afraid of the risks of smoking before surgery and ashamed for being a smoker, and that these feelings contributed to her smoking cessation.

So, I think that when they are doing the operation and they have planned this and they want me to quit if I could, then I thought that “Of course I need to give to this as well.”

Yes, but absolutely. And especially that feeling that I...when I walked out of that room, like both scared and ashamed and...it became “Yes, let’s do this.”

##### Perceptions of the Mobile Phone Medium

The data showed that users’ perceptions of the mobile phone medium and the text messages used to reach them contributed to how they used the intervention and how they appraised the content. Respondents described that text messages as a medium to deliver the intervention was useful, reliable, and required little from them as users. For example, one respondent perceived text messages to be more personal than mobile apps, which were thought of as more generic. Another respondent expressed that text messages gave a more serious impression than other methods, such as social media.

So, it becomes like there is somebody that sits there and sends the message, or maybe they maybe it is automated, but you take it seriously ‘cause it comes as a text message.

Informants expressed that receiving cessation support and factual information on the phone, in the text message format, was described as valuable and that the content, albeit something they already knew, was thought about in a different way when it arrived via their personal phone.

I don’t care what it says there [health adverts campaigns], but ‘cause it was in a text message...yes, then it becomes more personal so you take it in a different way.

However, although respondents described that they read most of the text messages, they expressed that they engaged very little with the interactive modules of the intervention. Respondents described that using the intervention had to be effortless.

And then it was like it was quite effortless for me, I don’t need to do that much, but that there was somebody else all the time that reminded me or motivated me; do you know what I mean?

I didn’t want to sit with it [mobile phone]; I thought that it was so awkward with it. No, so...no, I didn’t, I never...yes, that’s what it’s, it’s awkward to sit. But I thought reading the text message, like, that I found ok.

## Discussion

### Principal Findings

This study investigated the implementation of facilitated access where smoking cessation specialists invited patients to sign up for the intervention during a face-to-face visit. Also, the perceived usefulness of the text messaging, smoking cessation intervention among patients waiting to undergo surgery was explored. Smoking cessation specialists used strategies such as building trust with patients and adapting their approach to drive the implementation forward. Specialists perceived the intervention as a useful complement to routines and that its content and structure were compatible with existing practice. Findings from interviews showed that this patient group had a strong motivation to quit smoking and that the timing of the intervention influenced how text messages were perceived and used. Patients’ use of the intervention was characterized by an openness to learn, embracing the advice given, and humanizing and sharing of text messages. The intervention was often used as a complement, in combination with other strategies such as physical activity.

Findings showed that smoking cessation specialists found the facilitated access elements relatively straightforward to implement into their routines. Specialists talked about how they recognized the content and advice given in the intervention to be compatible with their existing practice. Also, they perceived the intervention to be a valuable and useful complement to existing routines. Implementation theory and research propose that *how* a new practice is perceived among key stakeholders is important for how, and to what extent, it is implemented [32]. Accordingly, how users perceive the characteristics of an intervention in terms of its compatibility, trialability, complexity, relative advantage, and observability will contribute to how the intervention is used and implemented. Smoking cessation specialists expressed that the content of the intervention was compatible with other strategies that were used and that the intervention could fit into the session structure. However, other aspects, such as perceived complexity of the intervention and its implementation, relative advantage, and observability of the benefits of the intervention, could have made implementation more difficult. Findings do not indicate that facilitated access was difficult to carry out, per se; however, specialists expressed that they struggled to communicate the intervention to patients in an optimal way and that limited time made it challenging to always engage patients. Relative advantage and observability are difficult to pinpoint, as the intervention was thought of, and intended, as a complement to other support. Thus, implementing facilitated access of a text message intervention in routine smoking cessation practice seemed to have been facilitated by characteristics such as the perceived compatibility; however, implementation could have been further facilitated in terms of how the intervention was communicated to patients.

Furthermore, findings showed that specialists overcame barriers to implement by adapting how they presented and spoke about the intervention as well as which role they adopted, depending on the specific need of the situation or patient. Implementation theory proposes that innovations that can be adapted to fit local needs and resources are easier to implement. The notion of adapting evidence-based programs and interventions was defined decades ago as the degree to which an intervention is altered in the process of adoption and implementation [32]. Recent definitions highlight in what way adaptations occur, such as the content and delivery of an intervention [33,34]. Previous research has shown that adaptations occur due to the needs and situations of patients or practitioners [35], due to the restraints such as limited time or resources [35,36], to promote recruitment or retention [37], and to increase the fit between the intervention and the actual implementation setting [35]. A potential challenge with text message–based interventions, that are generic and automated, is that they can seldom be tailored to individual patients compared to, for example, motivational interviewing. However, our findings showed that the routines around the intervention and how practitioners talked about the intervention was adapted to the situation.

Moreover, adapting how the intervention was presented to patients meant that specialists took a relatively active role in the implementation process. The fact that they built trust with patients to contribute to introducing the intervention is another example of adopting an active role and taking responsibility for implementation. Interestingly, specialists used existing therapy strategies to enable and optimize the conditions for implementation (ie, building trust with patients). This suggests that facilitated access could be a useful setting for implementation, as it is facilitated by the patient-practitioner relationship. The way specialists approached implementation and their flexibility most probably facilitated implementation efforts.

Furthermore, the health situation of the patients could have promoted implementation and use of the intervention. The findings illustrated a patient group that was relatively engaged in both their behavior change and using the intervention. Patients were receptive of the intervention, which can be illustrated by their openness toward the content of the text messages and sharing of the intervention with significant others. However, how text messages were thought of and humanized indicate that the patients processed and integrated the content of the messages into their behavior change efforts. Self-determination theory proposes different types of motivations that are driven predominantly either by controlled conditions (eg, external motivation, where behavior change happens due to someone telling you to change) or autonomous conditions (eg, intrinsic motivation, where behavior change is rewarding in itself) [38]. Our findings suggested that the patient group expressed external motivation rather than intrinsic motivation. For instance, patients described that requirements to quit smoking before surgery and to optimize healing postsurgery had prompted and motivated them to quit smoking. Behavior change based on external motivation, rather than intrinsic motivation, is more difficult to sustain in the long term. Thus, smoking cessation before surgery could be an effective time to reach smokers; however, interventions should ensure that cessation persists is the long term.

However, the interviews also showed that usage was limited regarding interactive modules and depended on the components being effortless and on the timing, of both the intervention and the content, being optimal. The role of timing was highlighted by both specialists and patients. Patients stressed the importance of timing between their quit date and commencing the intervention. Practitioners suggested that identifying and recruiting patients would be more efficient in primary care rather than waiting for the surgery department to refer patients. Previous research has highlighted that preoperative interventions are indeed difficult to implement and that primary care is an ideal setting for implementing smoking cessation [39]. This has implications for future implementation efforts of similar interventions in terms of when to introduce similar interventions for smoking cessation support in health care organizations.

### Methodological Considerations

A limitation of the study is the number of focus groups and interviews that were carried out. The study had access to two smoking cessation offices that, in total, employed three smoking cessation specialists. Although all the specialists were invited to focus groups, this is a limited number. In addition, only women took part in the focus groups, which could have been a potential limitation. All specialists that worked at the offices were invited to take part in focus groups and all were women. This may represent the real-world situation of smoking cessation and nursing practice, which includes professional contexts where women are overrepresented. Nevertheless, a limited number of men in the study could have been a limitation.

Credibility of the study could have increased by including additional hospitals. Unfortunately, this was not feasible within the time frame of the study. However, we believe that the findings are still valuable in illustrating how a text messaging, smoking cessation intervention can be implemented and used in this kind of setting. We also tried to endorse credibility by involving different researchers in the data collection (CL, PB, and KT), main data analysis (KT), and critical review of the main findings (all authors). The trustworthiness of the main findings in terms of credibility was reviewed by all authors. Trustworthiness of the study in terms of dependability was increased by using data from two perspectives (ie, specialists and patients), using interview guides for all data collection, and employing a structured and systematic data analysis process. Interestingly, findings from the two datasets verified each other, for example, regarding the importance of timing and the use of the intervention as a complement. Reflexivity was used during the data analysis to increase confirmability. This was done by critically reviewing the analysis process (eg, rationale for merging codes and creating categories). Confirmability could have been increased further, however, through also systematically using reflexivity during the data collection phase and creating an explicit audit trail depicting the whole research process. Finally, the findings could be transferable to smoking cessation counseling practice in hospital settings and among adult patient preoperative populations.

### Implications

The findings showed that preoperative patients exhibit strong motivation for smoking cessation and an openness toward using their mobile phones in their efforts to quit smoking. These findings imply great potential for health care systems to incorporate digital tools in practice to support patients. However, more research is needed that investigates the impact of different implementation strategies. This study focused on facilitated access at a smoking cessation unit in a hospital setting; future studies could explore implementation in primary care settings. Indeed, the findings highlighted the importance of timing between patients’ quit dates and the intervention start date. By exploring possibilities of reaching patients in primary care, the timing could potentially be improved.

### Conclusions

Smoking cessation specialists adopted an active role in implementing the intervention by adapting their approach and fitting the intervention into existing routines. Patients showed strong motivation to quit smoking and an openness to incorporate the intervention into their behavior change journey; however, the timing of the intervention and messages were important to optimize support. A text messaging, smoking cessation intervention can be a valuable and feasible way to reach smoking patients having elective surgery.

## Abbreviations

NEXit: Nicotine Exit
